# Accuracy of delirium risk factors in adult intensive care unit patients

**DOI:** 10.1590/1980-220X-REEUSP-2021-0222

**Published:** 2022-01-05

**Authors:** Luciana Aparecida Costa Carvalho, Marisa Dibbern Lopes Correia, Ráisa Camilo Ferreira, Micnéias Lacerda Botelho, Elaine Ribeiro, Erika Christiane Marocco Duran

**Affiliations:** 1 Universidade Estadual de Campinas, Faculdade de Enfermagem, Programa de Pós-Graduação em Enfermagem, Campinas, SP, Brazil.; 2 Universidade Federal de Viçosa, Departamento de Medicina e Enfermagem, Viçosa, MG, Brazil.; 3 Universidade Federal de Mato Grosso, Campus Sinop, Cuiabá, MT, Brazil.

**Keywords:** Delirium, Risk Factors, Hospitalization, Intensive Care Units, Data Accuracy, Delirio, Factores de Riesgo, Hospitalización, Unidades de Cuidados Intensivos, Exactitud de los Datos, Delírio, Fatores de Risco, Hospitalização, Unidades de Terapia Intensiva, Confiabilidade dos Dados

## Abstract

**Objective:**

To assess the accuracy measurements for predisposing and precipitating Risk Factors for delirium in an adult Intensive Care Unit.

**Method:**

Cohort, prospective study with patients over 18 who had been hospitalized for over 24 hours and were able to communicate. The patients were assessed once a day until the onset of delirium or permanence in the Intensive Care Unit. Instruments were employed to track delirium, characterize the sample, and identify the risk factors. Descriptive statistics was employed for sample characterization and accuracy tests for risk factors.

**Results:**

The included patients amounted to 102, 31 of which presented delirium. The predisposing predictive risk factors were hypoalbuminemia, American Society of Anesthesiology over three, severity, altered tissue perfusion, dehydration, and being a male, whereas precipitating predictive factors were physical restraint, infection, pharmacological agent, polypharmacy, anemia, altered renal function, dehydration, invasive devices, altered tissue perfusion and altered quality and quantity of sleep.

**Conclusion:**

An accurate identification of predisposing and precipitating risk factors may contribute to planning preventive measures against delirium.

## RESUMO

**Objetivo:**

Avaliar as medidas de acurácia dos Fatores de Risco predisponentes e precipitantes para o *delirium* em pacientes em Unidade de Terapia Intensiva adulto.

**Método:**

Estudo de coorte prospectivo em pacientes maiores de 18 anos, mais de 24 horas de internação, capazes de se comunicar, avaliados uma vez ao dia, até desenvolvimento do *delirium* ou permanência na Unidade de Terapia Intensiva. Utilizou-se instrumentos para rastreio do *delirium*, caracterização da amostra e identificação dos fatores de risco. Estatística descritiva para caracterização da amostra e testes de acurácia aos fatores de risco.

**Resultados:**

Incluídos 102 pacientes e 31 apresentaram *delirium*, identificado fatores de risco preditivos predisponentes: hipoalbuminemia, *American Society of Anestesiology* maior que três, gravidade, alteração da perfusão tissular, desidratação e sexo masculino, já os preditivos precipitantes: restrição física, infecção, agente farmacológico, polifarmácia, anemia, alteração da função renal, desidratação, dispositivos invasivos, alteração da perfusão tissular e alteração na qualidade e quantidade do sono.

**Conclusão:**

Identificação acurada dos fatores de risco predisponentes e precipitantes podem auxiliar no planejamento de medidas preventivas ao *delirium*.

## RESUMEN

**Objetivo:**

Evaluar las medidas de exactitud de los factores de riesgo predisponentes y precipitantes del delírium en pacientes de una Unidad de Cuidados Intensivos de adultos.

**Método:**

Estudio de cohorte prospectivo en pacientes mayores de 18 años con más de 24 horas de hospitalización y capaces de comunicarse, evaluados una vez al día hasta el desarrollo de delírium o estancia en la Unidad de Cuidados Intensivos. Se utilizaron instrumentos para seguir el delírium, caracterizar la muestra y identificar los factores de riesgo. Se utilizaron estadísticas descriptivas para caracterizar la muestra y pruebas de exactitud para los factores de riesgo.

**Resultados:**

Se incluyeron 102 pacientes y 31 presentaron delírium. Se identificaron los factores de riesgo predictivos predisponentes: hipoalbuminemia, puntuación de la *American Society of Anesthesiology* superior a tres, gravedad, cambios en la perfusión tisular, deshidratación y sexo masculino. Los factores de riesgo precipitantes fueron: restricción física, infección, agente farmacológico, polifarmacia, anemia, cambio de la función renal, deshidratación, dispositivos invasivos, cambios en la perfusión tisular y cambios en la calidad y cantidad del sueño.

**Conclusión:**

La identificación exacta de los factores de riesgo predisponentes y precipitantes puede ayudar a planificar medidas preventivas contra el delírium.

## INTRODUCTION

Delirium is described as a disturbance in attention, conscience, and cognition, with a brief period of development, oscillating in severity throughout the day; it is related to physiological changes in the individual^(1)^. Its physiopathology is not completely established and the main hypothesis refers to a change in the concentration of neurotransmitters such as acetylcholine, serotonin, dopamine, melatonin, noradrenaline, and gamma-Aminobutyric acid (GABA). The increased secretion of cytokines and its high release in chronic stress result in an inflammation and increase the permeability of the blood-brain barrier, changing neurotransmission. Delirium is thus a likely result of different pathogenetic mechanisms, which may lead to reduced oxidative metabolism of the brain^(2)^.

The incidence of delirium varies according to the studied population. It is identified in up to 83.3% of patients in mechanical ventilation (MV) in general Intensive Care Units (ICU) and corresponds to the most frequent neurological dysfunction^(3)^. It is pointed as responsible for an increased time of hospitalization at the ICU, functional decline, higher institutionalization, long-lasting hospitalization, loss of invasive devices, higher costs, and higher mortality rate^(4)^.

To track delirium in the ICU, nurses may use the Confusion Assessment Method Intensive Care Unit (CAM-ICU), conceived for patients in severe state or in MV, with an overall sensitivity of 72.5% and specificity of 96.2% for Brazilian Portuguese^(5)^. In addition to its identification, preventive actions must be prioritized to decrease the risk of developing delirium, given its deleterious consequences to patients and the health system. Understanding the factors leading to its development is thus essential in this process^(4,6)^.

Delirium may be triggered by only one Risk Factor (RF) but is frequently considered a multifactorial condition. In most cases, there is an inter-relation between predisposing (vulnerability of the individual) and precipitating factors (damaging events during hospitalization)^(6)^.

Some RF are described in the literature, such as: being over 65 years old, patient severity, smoking, drinking, hypertension, dehydration, previous cognitive impairment, high number of hospitalization days, use of sedatives and analgesics, mechanical restriction, invasive devices, MV, and pain^(6–11)^. However, the nurse must identify the most accurate for their patients’ profile, with an emphasis on the RF of independent performance, providing early aid to the determination of preventive interventions. In this context, the primordial work of the nursing team is emphasized, since it maintains bedside surveillance, which enables an early identification of RF^(4,6)^.

The use of accuracy measures to determine the RF whose diagnosis development is most likely shows the representation of each factor regarding delirium as a phenomenon, directing the nursing practice to be based on scientific evidence^(12)^. That said, the nurse, who is responsible for planning patient care, must identify the priority interventions for solving nursing problems. Recognizing RF related to delirium is insufficient and identifying those which are more accurate for the outcome is a necessity. To this moment, there are no studies which provide accuracy between predisposing and precipitating RF for the development of delirium^(4,6)^.

In this context, this study aims to assess the accuracy measures of predisposing and precipitating RF of delirium in adult ICU patients. The expectations are thus to identify the RF to subsidize the nurses’ future planning of care to patients with susceptibility to delirium.

## METHOD

### Design of Study

Prospective cohort study guided by the instrument Standards for Reporting Studies of Diagnostic Accuracy (STARD)^(13)^.

### Local

This study was performed in the ICU of a university hospital in São Paulo state’s inland, Brazil. This ICU had 410 inpatient beds, 51 of which for the adult ICU.

### Sample Definition

The population comprised patients hospitalized in the Adult ICU for over 24 hours. The sample was obtained by convenience for a collection time of five months (September 2018 to January 2019).

### Selection Criteria

Patients over 18 years old capable of responding to CAM-ICU verbally or through gestures were randomly included. Excluded patients were those who, due to a change in their clinical conditions, were unable to respond to the items of this instrument^(5)^. For statistical analysis, patients who developed delirium during the assessment period and those who did not were included.

### Data Collection

The participants were assessed once a day by the researchers. The main researcher was responsible for clinical assessments, whereas the other researchers were responsible for medical record data collection. This was performed daily (morning, afternoon, or evening) from September 2018 to January 2019. These cases were followed up until the development of delirium. Patients who did not present this outcome continued to be assessed until ICU discharge, death, or transference. The main researcher performed case studies suggested by CAM-ICU prior to starting clinical assessments.

In addition, an instrument elaborated by the researchers for data collection was employed. This aimed at characterizing the population and identifying the RF, as well as their conceptual definitions (theoretical meaning) and operational definitions (how a given concept is applied and measured in practice). To identify all RF available in the literature, regardless of the clinical profile of the assessed patient, an Integrative Literature Review (IR) was performed, including: the phases of theme identification, literature search, study categorization, assessment of included studies, interpretation of results, and synthesis of knowledge available in the analyzed articles^(14)^.

The content of this instrument is emphasized to have been analyzed and appreciated by three judges of the Study and Research Group of Nursing Care Technologies (*Grupo de Estudos e Pesquisa sobre Tecnologias do Cuidar em Enfermagem*). The instrument includes information about current medical diagnosis, clinical history, sex, age, and marital status. Through medical record consultation, the following RF were obtained: patient severity (obtained through Acute Physiology and Chronic Health Evaluation (APACHE), which predicts hospital mortality, and through Sequential Organ Failure Assessment (SOFA), which assesses the degree of organ dysfunction and patient permanence in the ICU)^(7,15)^; infection (leukocyte values^(16)^; C-reactive protein value^(17)^; systolic blood pressure; respiratory frequency; temperature and Glasgow scale^(18))^; polypharmacy (number of medications administered in the previous 24 hours)^(19)^; altered renal function (creatinine, urine volume in 24 hours and glomerular filtration rate – GFR)^(16,20)^; time of the anesthetic and surgical procedure^(21)^; pharmacological agent (number and pharmacological class of the medication administered in the previous 24 hours)^(7,22)^; days of ICU hospitalization (days between admission and outcome)^(11)^; history of delirium (identification of the medical diagnosis in the medical record)^(23)^; dehydration (urea nitrogen/creatinine)^(9,16)^; nutritional deficiency (acceptance of nutrition per oral route, administration of enteral diet or prescribed parenteral nutrition)^(23)^; altered tissue perfusion (mean blood pressure^(21)^; history of systolic blood pressure^(8,11)^; diabetes mellitus^(20,23)^; cerebrovascular accident^(7,10–11)^ or brain hemorrhage^(11)^); blood transfusion^(24)^; American Society of Anesthesiology (ASA) over three^(21)^; anemia (hemoglobin – Hb value)^(24)^; dementia^(7,21)^; hypoalbuminemia (albumin value)^(25)^; male^(20)^; 60 years old or higher^(7–8,21)^.

The following were obtained through clinical assessment or direct interview with the patients: MV – considered as artificial ventilation the application of positive pressure on the airways, which may be invasive or non-invasive^(7,11)^; physical restraint (mechanical restriction to bed, multiparametric monitoring, prescribed movement inhibition with or without orthotics and external fixators)^(7)^; functional impairment (Barthel index)^(7,9)^; pain (visual analog pain scale)^(11)^; invasive devices (number and types of catheters and drains)^(7,19)^; altered visual acuity (use of glasses or contact lenses, total vision loss or report of difficulty to see)^(6)^; alcohol abuse (volume and type of consumed alcoholic beverage)^(6,21)^, smoking (reporting having smoked one or more days in the last 30 days)^(6,8)^; altered quantity and quality of sleep (how they slept and resting sensation)^(7)^ and comorbidity (Charlson index)^(26)^.

After statistical analysis and for a better comprehension, the RF were subdivided into predisposing and precipitating. During the phase of daily patient follow-up, for identification of delirium, CAM-ICU was employed. This instrument assesses the four patient features: 1 – “fluctuating mental status”; 2 – “inattention”; 3 – “disorganized thinking”; and 4 – “altered level of consciousness”. For a positive assessment of delirium, the presence of features one, two, and three or one, two, and four were considered, according to present or absent response pattern by the patient. The mean time of assessment was five minutes^(5)^.

### Data Analysis and Treatment

The data were stored in Microsoft Excel^®^ spreadsheets for the application of descriptive statistics to characterize the sample and to obtain frequencies, measures of central tendency (mean, median, minimum, and maximum) and dispersion (standard deviation – SD)^(12)^. The accuracy of the RF was assessed through calculation of measures of Sensitivity (SE), Specificity (SP), Positive Predictive Value (PPV), Negative Predictive Value (NPV), Positive Likelihood Ratio (LR+), Negative Likelihood Ratio (LR-), and Diagnostic Odds Ratio (DOR)^(12,27)^.

The test’s SE refers to the probability of it being positive in the presence of the outcome; SP refers to the probability of the test being negative in the absence of the outcome; PPV is the probability of the outcome when the test is positive; NPV is the probability of absence of the outcome when the test is negative. The LR+ refers to the probability of a result being positive for sick individuals over the probability of the result being positive for healthy individuals; LR-refers to the probability of a negative result for sick individuals over the probability of the result being negative for healthy individuals; DOR represents the chance of occurrence of the outcome among those exposed divided by the chances of the outcome for the non-exposed^(12,27)^.

To determine the accuracy, the test’s SE higher than 0.6 was adopted, given that there is no appropriate theoretical framework for its cut point, LR+ over one, LR-lower than one, and DOR over one. The more sensitive a factor is, the higher the chances of the event in the presence of a RF. Thus, considering the high variation of delirium incidence, this cut point was opted for; in this point, the chance of development of delirium is higher than that of no development in the presence of a given RF^(27)^. When sensitivity was equal to 1.00, the numerator was zero, and thus the result could not be calculated.

Finally, all analyses considered a 5% significance level. The analysis was assisted by statistical software Statistical Analysis Software^®^ version 9.4 and Statistical Package for the Social Sciences^®^ 22.0.

### Ethical Aspects

The study abided by the Brazilian National Health Council’s Resolution n. 466/12, referring to research involving human beings, and was approved in 2018 by the Ethics Committee of *Universidade Estadual de Campinas* on opinion 2.502.946. To start the assessments, the patient and/or responsible were previously approached for research clarifications and so that signature of the Informed Consent Form (ICF) could be requested. The assessments were started only upon authorization.

During data collection, the patient was approached and oriented concerning the procedure on all occasions. When delirium or any complaint was identified, it was reported to the health team in charge of the patient’s care.

## RESULTS

The study included 102 patients during the data collection period. No individuals were excluded. Among the included patients, 31 (30.4%) presented delirium. The ones who did not were assessed until ICU discharge, death, or transference, with a mean of 7.4 days of assessment per individual, in addition to a mean age of 54 with a SD of 15.4. The other sociodemographic data are described in Table 1.

**Table 1.** Epidemiological characterization of the patients included in the study per sex, age, primary cause of hospitalization, and type of treatment. Campinas, SP, Brazil, 2021. (n = 102).

Variable	n (102)	%
**Sex**		
Female	47	46.0
Male	55	54.0
**Age**		
18 to 29 years	8	7.8
30 to 59 years	43	42.2
Over 60 years	51	50
**Primary cause of hospitalization**		
Others	4	3.9
Abdominal	5	4.9
Renal	6	5.9
Respiratory	6	5.9
Trauma	13	12.7
Neurological	17	16.7
Cardiac	51	50.0
**Type of treatment**		
Clinical	41	40.2
Surgical	61	59.8

Other causes of hospitalization referred to myasthenia gravis, porphyria, diabetic ketoacidosis, and grade IV hepatic encephalopathy. Among the RF identified in the literature, it was not possible to assess dementia, since this diagnosis was not in the analyzed medical records. In this study, altered renal function was analyzed only as a precipitating factor, given that the obtained data were not sufficient to differentiate this alteration between chronic and acute.

The accuracy measures of the predisposing RF are presented in Table 2.

**Table 2.** Accuracy measures of RF predisposing to the development of delirium regarding sensitivity, specificity, positive predictive value, negative predictive value, positive likelihood ratio, negative likelihood ratio, and diagnostic odds ratio. Campinas, SP, Brazil, 2021. (n = 102).

RF*	SE^†^	SP^‡^	PPV^§^	PPN^||^	^#^LR+^$^(CI)	^+^LR-^$^(CI)	^##^DOR^$^(CI)
Hypoalbuminemia	1.00	0.36	0.58	1.00	1.58 (1.12; 2.23)	–	–
ASA > 3**	0.87	0.55	0.47	0.90	1.98 (1.47; 2.65)	0.22 (0.09; 0.57)	8.81 (2.79; 27.78)
Severity	0.81	0.64	0.50	0.88	2.28 (1.59; 3.25)	0.29 (0.14; 0.61)	7.80 (2.83; 21.49)
Altered tissue perfusion	0.75	0.40	0.36	0.77	1.25 (0.95; 1.65)	0.62 (0.32; 1.22)	2.00 (0.79; 5.08)
Dehydration	0.71	0.60	0.45	0.82	1.80 (1.25; 2.57)	0.47 (0.26; 0.84)	3.83 (1.55; 9.49)
Male	0.62	0.50	0.36	0.74	1.25 (0.88; 1.78)	0.75 (0.45; 1.24)	1.67 (0.71; 3.92)
Altered visual acuity	0.53	0.28	0.25	0.57	0.74 (0.52; 1.06)	1.64 (0.97; 2.77)	0.45 (0.19; 1.08)
Age > or equal to 60 years old	0.50	0.51	0.32	0.69	1.03 (0.67; 1.57)	0.97 (0.64; 1.47)	1.06 (0.46; 2.44)
Comorbidity	0.43	0.80	0.50	0.75	2.19 (1.19; 4.03)	0.70 (0.51; 0.98)	3.11 (1.25; 7.74)
Smoking	0.31	0.77	0.38	0.71	1.37 (0.70; 2.67)	0.89 (0.68; 1.16)	1.53 (0.60; 3.90)
Alcohol abuse	0.18	0.97	0.75	0.72	6.56 (1.40; 30.76)	0.84 (0.70; 0.99)	7.85 (1.49; 41.38)
Functional impairment	0.09	0.97	0.60	0.69	3.23 (0.57; 18.42)	0.93 (0.83; 1.05)	3.47 (0.55; 21.85)
History of *delirium*	0.03	0.94	0.20	0.68	0.55 (0.06; 4.70)	1.03 (0.94; 1.12)	0.53 (0.06; 4.96)

*RF: Risk Factor; ^†^SE: Sensitivity; ^‡^SP: Specificity; ^§^PPV: Positive Predictive Value; ^||^NPV: Negative Predictive Value; ^#^LR+: Positive Likelihood Ratio; ^$^CI: Confidence Interval; ^+^LR-: Negative Likelihood Ratio; ^##^DOR: Diagnostic Odds Ratio and **ASA: American Society of Anesthesiology.

The RF alcohol abuse, functional impairment, and history of delirium had a high specificity, showing that, in the absence of these factors, delirium has a 94–97% probability of not being present.

The accuracy measures of the precipitating RF are presented in Table 3. The analysis of the RF “invasive devices” identified a mean of two devices per individual. The analysis was thus built based on this result.

**Table 3.** Measures of accuracy and of the precipitating RF for the development of delirium regarding sensitivity, specificity, positive predictive value, negative predictive value, positive likelihood ratio, negative likelihood ratio, and diagnostic odds ratio. Campinas, SP, Brazil, 2021. (n = 102).

RF*	SE^†^	SP^‡^	PPV^§^	PPN^||^	^#^LR+^$^(CI)	^+^LR-^$^(CI)	^##^DOR^$^(CI)
Physical restraint	1.00	0.05	0.32	1.00	1.06 (1.00; 1.12)	–	–
Infection	0.96	0.37	0.41	0.96	1.55 (1.28; 1.89)	0.08 (0.01; 0.58)	18.74 (2.41; 145.6)
Pharmacological agent	0.96	0.05	0.31	0.80	1.03 (0.94; 1.12)	0.55 (0.06; 4.70)	1.88 (0.20; 17.52)
Polypharmacy	0.93	0.10	0.32	0.77	1.04 (0.93; 1.17)	0.62 (0.14; 2.84)	1.67 (0.33; 8.51)
Anemia	0.84	0.21	0.33	0.75	1.08 (0.89; 1.31)	0.72 (0.29; 1.81)	1.50 (0.49; 4.56)
Days of ICU	0.81	0.18	0.31	0.68	1.00 (0.82; 1.22)	1.01 (0.42; 2.42)	0.99 (0.34; 2.89)
Altered renal function	0.78	0.48	0.40	0.82	1.52 (1.13; 2.03)	0.45 (0.22; 0.90)	3.37 (1.29; 8.81)
Dehydration	0.71	0.60	0.45	0.82	1.80 (1.25; 2.57)	0.47 (0.26; 0.84)	3.83 (1.55; 9.49)
Invasive devices (>2)	0.75	0.87	0.72	0.88	5.83 (3.07; 11.08)	0.29 (0.16; 0.53)	20.33 (7.02; 58.87)
Altered tissue perfusion	0.75	0.40	0.36	0.77	1.25 (0.95; 1.65)	0.62 (0.32; 1.22)	2.00 (0.79; 5.08)
Altered quality and quantity of sleep	0.68	0.48	0.37	0.77	1.34 (0.96; 1.85)	0.64 (0.36; 1.13)	2.08 (0.86; 5.02)
Time of the anesthetic and surgical procedure	0.61	0.18	0.17	0.64	0.76 (0.48; 1.19)	2.05 (0.83; 5.07)	0.37 (0.10; 1.40)
Nutritional deficiency	0.46	0.52	0.31	0.68	0.99 (0.64; 1.55)	1.01 (0.68; 1.49)	0.99 (0.43; 2.29)
Mechanical ventilation	0.43	0.95	0.82	0.78	10.21 (3.15; 33.05)	0.59 (0.43; 0.80)	17.37 (4.50; 67.08)
Pain	0.28	0.77	0.36	0.70	1.23 (0.62; 2.48)	0.93 (0.72; 1.20)	1.32 (0.51; 3.42)
Blood transfusion	0.03	0.98	0.50	0.69	2.19 (0.14; 33.88)	0.98 (0.92; 1.05)	2.23 (0.13; 36.75)

*RF: Risk Factor; ^†^SE: Sensitivity; ^‡^SP: Specificity; ^§^PPV: Positive Predictive Value; ^||^NPV: Negative Predictive Value; ^#^LR+: Positive Likelihood Ratio; ^$^CI: Confidence Interval; ^+^LR-: Negative Likelihood Ratio and ^##^DOR: Diagnostic Odds Ratio.

The RF blood transfusion and MV presented a high specificity, showing that, in the absence of these RF, delirium has a risk of not being present in 95–98% of cases. After the identification of the accuracy of the RF pharmacological agent, each one was analyzed separately (Proton-pump inhibitors, Analgesics, Opioid analgesics, Moderate cholinesterase inhibitor, Antipsychotics, Corticosteroids, Hypnotics/Anxiolytics, Very strong cholinesterase inhibitor, Antidepressants, and General anesthetics). Only proton-pump inhibitors (SE – 0.6875) and analgesics (SE – 0.6875) were predictive for the development of delirium. No patient with delirium received general anesthetics during the period of assessment, and the sensitivity of this item was thus zero.

The RF “invasive devices” was analyzed separately and the Indwelling Bladder Catheter (SE – 0.8750) was the most predictive of the development of delirium, followed by the Central Venous Catheter (SE – 0.7500) and the Nasoenteral Catheter (SE – 0.7500).

## DISCUSSION

In this study, 30.4% of the patients presented delirium, which corroborates the incidence in the intensive care environment, which may be up to 83.3%^(3)^, being influenced by the characteristics of the population, presence of specific tracking methods, and preventive measures implemented for this outcome^(2,4–5)^. In the study unit, there were no systematic methods of delirium identification, risk, or conduction of preventive measures. The incidence found was within that described by the literature, particularly for a place with no specific measures for its management^(20)^.

This study’s sample was predominantly male, which is a predisposing factor described in the literature and predictive of delirium in this study^(20)^. Half of the included patients were 60 years old or older. In Brazil, the elderly population is defined by the World Health Organization as comprising individuals over 60 years old^(28)^. In this study, the RF age was not accurate for the development of delirium. However, old age may be related to neuronal apoptosis, reduction of brain blood flow and alteration in the neurotransmitter system, elucidating from a physiological point of view its relationship in other works discussing increased risk of delirium^(21)^.

The main cause of hospitalization was cardiac and the most incident chronic disease in Brazilians is cardiovascular disease, which justifies this result^(29)^. Associated to that, surgical treatment was the most conducted one, since 25 of the investigated beds were postoperative and some patients of the coronary and clinical unit were not exempt of possible surgical interventions during hospitalization.

The predisposing RF hypoalbuminemia presented a significantly lower value in patients with delirium, which makes it possible to state its relation with the outcome. This may indicate a poor nutritional state prior to hospitalization, loss of proteins through urine or alteration of its production by the liver. Thus, as with the resulting reduction of osmotic force, there is a difficulty in the permanence of the intravascular volume, which may lead to a reduction in brain perfusion. In addition, the proteins are responsible for transportation of some medication, enabling its free concentration in plasma to become high, increasing the risk of delirium^(25)^.

The subjective measure of comorbidities and the preoperative patient conditions may be assessed through ASA, in which a score higher than three is predictive of delirium^(21)^. A higher number and severity of previous patient comorbidities – which cause chronic altered tissue perfusion –, smaller events during hospitalization which intensify this alteration, may be determinant for the reduction of brain perfusion and the development of delirium^(21)^.

Patient severity, defined as intensity and extension of organic dysfunction and of the disease presented by the individual, which influences prognosis, was identified through an APACHE score over 16 and SOFA higher than or equal to five^(7,15)^. This RF presented a high predictive power for delirium in this study, probably due to the lower physiological reserve of the individual due to condition severity. Thus, in this context, the presence of a lower number of precipitating RF is necessary for the development of delirium^(6)^.

In addition to that, a reduction in blood flow of tissues that compromise health may be identified through a previous diagnosis of Systemic Arterial Hypertension, Diabetes Mellitus, Mean Blood Pressure lower than 55 mmHg, Cerebrovascular Accident, or brain hemorrhage, leading to an alteration in tissue perfusion^(7–8,10–11,20,21,23)^. These parameters must be analyzed in the moment of hospitalization and related to the likely alteration in brain tissue perfusion during hospitalization.

Also, dehydration, i.e., the reduction of the extracellular volume secondary to hydroelectrolytic loss, identified through a urea nitrogen/creatinine ratio over 18, was also a predisposing RF. This was probably due to a contraction of intravascular volume, reduction of tissue perfusion, and global reduction of the mechanism of brain oxidation^(2,9)^. This factor may be identified upon patient admission, becoming a predisposing or precipitating RF during hospitalization.

Therefore, preventive measures for these factors must be targeted at therapeutic measures with the objective of reducing cognitive decline, sensory loss, and an adequate hydration and nutrition. In addition, the assessment of the presence of relatives in the ICU must be considered, since it provides higher sensory stimulus known by the patient, in addition to reducing cognitive decline^(2,6)^.

The precipitating RF physical restraint, validated in this study, may be caused by patient stress and reduction of sensory stimulation known by the individual. In addition, mechanical restriction in bed must be the last resort to maintain safety and early mobilization is a preventive method^(2,6)^.

Similarly, the infection RF may be related to delirium, since inflammation during this process changes the permeability of the blood-brain barrier and, consequently, neurotransmission. For this reason, preventive actions against infection may also result in prevention of delirium^(2)^.

Proton-pump inhibitors and analgesics (all types of medication used for pain management) were the most predictive for the outcome. However, long-lasting use of proton-pump inhibitors still does not present a consistent relation with the development of delirium. The hypotheses include: (a) increased risk of infection (pneumonia and *Clostridium difficile*), which is a RF for delirium; (b) Vitamin B12 deficiency, which increases cognitive decline; (c) hypomagnesemia and interference in the pharmacokinetics of benzodiazepines and antidepressants, which may cross the blood-brain barrier^(22)^.

In addition, the administration of opioids may interfere in the regular working of the neurological system, particularly among the elderly. In these individuals, pharmacodynamics and pharmacokinetics of medications are altered by aging, which involves modifications in body composition and reduction of renal and liver function. Due to that, they are susceptible to more intense adverse or therapeutic effects, which may be related to the result obtained in this study^(21,28)^.

Consequently, polypharmacy is defined as the administration of five or more medications within 24 hours^(19)^ and was considered a precipitating predictive RF of delirium and its isolated value was significant for this outcome. This is due to the fact that it may be related to patient severity, which requires a high number of medications and/or stress caused by the disturbance of the constant manipulations needed for its administration^(2)^.

In addition, the reduction of hemoglobin compromises oxygen transportation to tissues, altering the brain oxidation system and increasing the risk of delirium. The RF Anemia was validated for this population. Thus, the correction of hemoglobin must be prioritized whenever they match the transfusion criteria^(2)^.

Altered renal function, defined as a condition when kidneys lose their capacity of performing basic functions, may be related to delirium due to accumulation of toxins (urea and creatinine) in the organism and consequent alteration in neurotransmission, including alteration in dopamine and serotonin. Both are identified in the physiopathology of delirium^(2,16,20)^.

Also, the presence of invasive devices was one of the precipitating RF most predictive of delirium and may be related to patient severity, increased risk of infection, and physical restraint. Thus, these devices must be removed whenever possible and this may occur through daily reassessments of its indication. In this study they were categorized according to their type and IDBC was the most predictive of this outcome, demonstrating a constant need for assessment of its indication^(6,16)^.

The lack of programmed care and excessive noise and light may be sources of stress for patients, alter the production of melatonin and quality and quantity of sleep, which may be altered in the presence of delirium. Thus, schedules must be planned for medication administration, vital signs measurement, and conduction of procedures for adequate sleep^(2)^.

Patients require thus actions which may provide time and space orientation. Such actions may be conducted through calendars, watches, familiar objects, reduction of noise and adequate illumination. Such factors provide a calm and comfortable environment. Reaffirming the presence of family is pointed out as a preventive measure against delirium, reducing patient stress and anxiety while promoting a better participation in care, improving connection with the environment^(2,6,30)^.

Concerning this study’s limitations, the number of patients included in each of the RF could have been higher; however, the low sample representation of some RF did not interfere in the outcome analysis. In addition to that, cognitive impairment was not assessed, since the high severity of this population did not allow for an objective assessment and the motor subtypes of delirium (hyperactive, hypoactive, mixed) were not identified, which precluded relating the RF with their motor subtypes.

Thus, identifying the most predictive RF for the development of delirium in a specific population has shown that not all factors identified in the literature are sensitive to the outcome. With that, the nurse needs to recognize the demand of the population, the accurate RF, and their physiological relation with delirium to direct specific and efficient nursing interventions.

The identification of these RF in this study may collaborate with planning and implementation of preventive actions for the studied population, particularly with the evidence of RF more predictive of delirium in the context of intensive care. In addition, this study shows that the RF identified in the literature are not always present in all populations and accuracy studies are needed to determine which are the most predictive for planning the nurses’ interventions.

Preventive actions against RF aim at the non-intensification of predisposing factors and prevention of occurrence of precipitating factors during hospitalization. The nurse plays thus a vital role in the early identification of these RF for subsequent care directing.

## CONCLUSION

In this study the predisposing RF most predictive of delirium were hypoalbuminemia, ASA over three, patient severity, altered tissue perfusion, dehydration, and being a male. Precipitating RF include physical restraint, infection, pharmacological agent, polypharmacy, anemia, altered renal function, dehydration, over two invasive devices, altered tissue perfusion, altered quality and quantity of sleep. Considering that tissue perfusion and dehydration are both predisposing and precipitating factors, the need for permanence of identification of these factors during hospitalization is emphasized.

## References

[1] American Psychiatric Association (2014). Manual diagnóstico e estatístico de transtornos mentais.

[2] Maldonado JR (2018). Delirium pathophysiology: An updated hypothesis of the etiology
of acute brain failure. Int J Geriatr Psychiatry..

[3] Ely EW, Inouye SK, Bernard GR, Gordon S, Francis J, May L (2001). Delirium in mechanically ventilated patients validity and
reliability of the confusion assessment method for the intensive Care Unit
(CAM-ICU). JAMA..

[4] Souza RClS, Bersaneti MDR, Siqueira EMP, Meira L, Brumatti DL, Prado NRO (2017). Nurses’ training in the use of a delirium screening
tool. Rev Gaucha Enferm..

[5] Gusmão-Flores D, Salluh JIF, Dal-Pizzol F, Ritter C, Tomasi CD, Lima MASD (2011). The validity and reliability of the portuguese versions of three
tools used to diagnose delirium in critically ill patients. Clinics.

[6] Devlin JW, Skrobik Y, Gélinas C, Needham DM, Slooter AJC, Pandharipande PP (2018). Clinical practice guidelines for the prevention and management of
pain, agitation/sedation, delirium, immobility, and sleep disruption in
adult patients in the ICU. Crit Care Med..

[7] Limpawattana P, Panitchote A, Tangvoraphonkchai K, Suebsoh N, Eamma W, Chanthonglarng B (2016). Delirium in critical care: a study of incidence, prevalence, and
associated factors in the tertiary care hospital of older Thai
adults. Aging Ment Health..

[8] Visser L, Prent A, van der Laan MJ, van Leeuwen BL (2015). Predicting postoperative delirium after vascular surgical
procedures. J Vasc Surg..

[9] Carrasco MP, Villarroel L, Andrade M, Calderón J, González M (2014). Development and validation of a delirium predictive score in
older people. Age Ageing..

[10] Lim TS, Lee JS, Yoon JH, Moon SY, Joo IS, Huh K (2017). Cigarette smoking is an independent risk factor for post-stroke
delirium. BMC Neurol..

[11] Kumar A, Jayant A, Arya V, Magoon R, Sharma R (2017). Delirium after cardiac surgery: A pilot study from a single
tertiary referral center. Ann Card Anaesth..

[12] Bossuyt PM, Reitsma JB, Bruns DE, Gatsonis CA, Glasziou PP, Irwig L (2015). STARD 2015: an updated list of essential items for reporting
diagnostic accuracy studies. BMJ.

[13] Pompeo DA, Rossi LA, Galvão CM (2009). Revisão integrativa: Integrative literature review: the initial
step in the validation process of nursing diagnoses. Acta paulista de enfermagem..

[14] Dessap AM, Roche-Campo F, Launay JM, Charles-Nelson A, Katsahian S, Brun-Buisson C (2015). Delirium and circadian rhythm of melatonin during waning from
mechanical ventilation: an ancillary study of wearing trial. Chest..

[15] De Castro SMM, Ünlü Ç, Tuynman JB, Honig A, Steller EP, Vrouenraets BC (2014). Incidence and risk factors of delirium in the elderly general
surgical patient. Am J Surg..

[16] Pol RA, van Leeuwen BL, Izaks GJ, Visser L, Tielliu IFJ, Zeebregts CJ (2014). C-reactive protein predicts postoperative delirium following
vascular surgery. Ann Vasc Surg..

[17] Smith PJ, Rivelli SK, Waters AM, Hoyle A, Durheim MT, Reynolds JM (2015). Delirium affects length of hospital stay after lung
transplantation. J Crit Care..

[18] Kim JY, Yoo JH, Kim E, Kwon KB, Han BR, Cho Y (2017). Risk factors and clinical outcomes of delirium in osteoporotic
hip fractures. J Orthop Surg..

[19] Fortini A, Morettini A, Tavernese G, Facchini S, Tofani L, Pazzi M (2014). Delirium in elderly patients hospitalized in internal medicine
wards. Intern Emerg Med..

[20] Guo Z, Liu J, Li J, Wang X, Guo H, Ma P (2017). Postoperative Delirium in Severely Burned Patients Undergoing
Early Escharotomy: Incidence, Risk Factors, and Outcomes. J Burn Care Res..

[21] Otremba I, Wilczyn’ski K, Szewieczek J (2016). Delirium in the geriatric unit: Proton-pump inhibitors and other
risk factors. Clin Interv Aging..

[22] van Eijsden WA, Raats JW, Mulder PG, van der Laan L (2015). New aspects of delirium in elderly patients with critical limb
ischemia. Clin Interv Aging..

[23] van der Zanden V, Beishuizen SJ, Scholtens RM, Jonghe A, de Rooij SE, van Munster BC (2016). The effects of blood transfusion on delirium
incidence. J Am Med Dir Assoc..

[24] Winangun MA, Aryana GPS, Astika N, Kuswardhani RAT (2020). Relationship of albumin serum levels and Neutrophil-Lymphocyte
Ratios (NLR) on activities of daily living elderly patients with delirium at
Sanglah General Hospital, Bali, Indonesia. Bali Med J..

[25] Ribeiro SCL, Nascimento ERP, Lazzari DD, Jung W, Boes AA, Bertoncello KC (2015). Knowledge of nurses about delirium in critical patients:
Collective subject discourse. Texto & contexto enfermagem..

[26] Lopes MVO, Silva VM, Araujo TL (2012). Methods for establishing the accuracy of clinical indicators in
predicting Nursing Diagnoses. Int J Nurs Knowl..

[27] Borges LSR (2016). Medidas de Acurácia Diagnóstica.. Int J Cardiovasc Sci.

[28] Oliveira HSB, Corradi MLG (2018). Pharmacological aspects of elderly: an integrative literature
review. Revista de medicina..

[29] Malta DC, Bernal RTI, Lima MG, Araújo SSC, Silva MMA, Freitas MIF (2017). Noncommunicable diseases and the use of health services: analysis
of the National Health Survey in Brazil. Rev Saude Publica..

[30] Contreras CCT, Páez-Esteban AN, Rincon-Romero MK, Carvajal RR, Herrera MM, Díaz del Castillo AH (2021). Nursing intervention to prevent delirium in critically ill
adults. Rev Esc Enferm USP..

